# Incidence of lymphedema related to various cancers

**DOI:** 10.1007/s12032-024-02441-2

**Published:** 2024-09-17

**Authors:** Marie-Eve Letellier, Marize Ibrahim, Anna Towers, Geneviève Chaput

**Affiliations:** Lymphedema Support Centre of the Quebec Breast Cancer Foundation at the MUHC, McGill University Health Centre, Montreal, QC Canada

**Keywords:** Cancer, Lymphedema, Incidence, Oncology, Survivorship, Long-term effects

## Abstract

Cancer-related lymphedema (CRL) lacks internationally accepted definition and diagnostic criteria. The accurate incidence of CRL is therefore a challenge and the condition is likely underreported. Patients treated for cancer can develop CRL as a result of surgery, chemotherapy, and/or radiotherapy, which can lead to considerable psychosocial and physical morbidity, and decreased quality of life. Determining CRL incidence is crucial to inform care access and resource allocation, to best support patients affected by this lifelong condition. This review aimed to provide the latest CRL incidence estimates. Using four core databases (MEDLINE, Embase, Web of Science Core Collection, Cochrane Library), a literature search was performed to capture publications dated between 2015 and 2023. A total of 48 articles (33 prospective studies, 15 systematic reviews) met inclusion criteria, providing a sample size of 234,079 cancer patients. Findings revealed CRL incidence across cancer types varied, reported 2–74% in breast, 8–45% in gynecological and urological, 71–90% in head and neck and 2–29% in melanoma cancers. CRL incidence varied between 3 and 21% in preventative lymphedema surgery patients. Projected increases in cancer incidence and improved survival rates are expected to further escalate CRL incidence. Healthcare systems and professionals alike must therefore prepare to meet the growing needs of CRL patients.

## Introduction

Cancer incidence and improved cancer survivorship have been increasing globally over the last few decades primarily due to population growth, aging populations, advances in cancer detection methods and changes in lifestyle and risk factors [1]. With an estimated 20 million new cancer cases reported annually worldwide, comes a plethora of long-term sequelae related to the cancers and their respective therapies, ranging from cardiotoxicity, functional impairments, cognitive issues, psychosocial and physical late and long-term effects, including cancer-related lymphedema (CRL) [2–4].

Cancer-related lymphedema is a chronic inflammatory process in the interstitial space due to reduced lymph transport capacity from damage to the lymph vessels, nodes or by direct tumor involvement [5–7]. CRL is progressive and may become a very debilitating condition for many cancer survivors, affecting the person physiologically, physically, and emotionally [8]. Because CRL requires lifelong management, cancer survivors often describe it as one of the most significant sequela of cancer treatment [2, 9, 10]. In the case of active cancers, malignant lymphedema may arise from infiltration, obstruction, or compression of lymphatic vessels and/or lymph nodes by the direct action of the tumor [11].

The impact of lymphedema on quality of life (QOL) can vary depending on the severity of the condition, its management, and the individual’s emotional, psychological, and physical well-being. Some of the extensively documented, significant effects of lymphedema on an individual’s QOL include physical discomfort, altered body image, reduced self-esteem, functional impairment, psychological distress, social barriers, occupational stressors, marginalization. In addition, patients may experience financial burdens because of costs associated with intensive treatment phases and lifelong expenses for compression garments [8, 12, 13]. Beyond these considerable individual impacts, there exists an economic strain on payers, healthcare systems, and society due to lymphedema-related hospitalizations for complications such as cellulitis and sepsis, as well as interruptions in employment [14–16].

Current estimates suggest that approximately 10 million individuals in the United States [17] and around 1 million in Canada [7] are affected by lymphedema. The etiology of lymphatic dysfunction could be primary, due to malformation of the lymphatic structures (vessels or nodes), or secondary, due to chronic lymphatic system overload (e.g., chronic venous insufficiency, obesity, CRL, trauma). Despite various causes of secondary lymphedema, with CRL being the most extensively documented, accurate estimates remain elusive due to inadequate documentation.

The reported incidence of CRL varies widely depending on cancer type, associated treatments and individual variability. Several factors contribute to the complexity of estimating CRL incidence, including the absence of a standardized international definition of lymphedema (e.g., definition based on varied clinical volume differences, self-report of swelling), variability in diagnostic assessment methods and criteria (e.g., circumference measurements—anatomical landmarks, every 10 cm; perometry), lack of pre- and post-operative screening protocols, and inconsistencies in timing and duration of follow-up assessments.

To varying degrees, CRL may affect many cancer survivors who have undergone node dissection surgery, chemotherapy, and/or radiotherapy. In this review, we expand upon the groundwork laid by Cormier et al. (2010) [9] and Shaitelman et al. (2015) [18] to provide updated insights into the incidence of CRL across a wide range of cancers. Estimating CRL incidence is crucial to inform care access and resources allocation needs to best support patients affected by this lifelong condition.

## Methods

### Search strategy

**Fig. 1:**
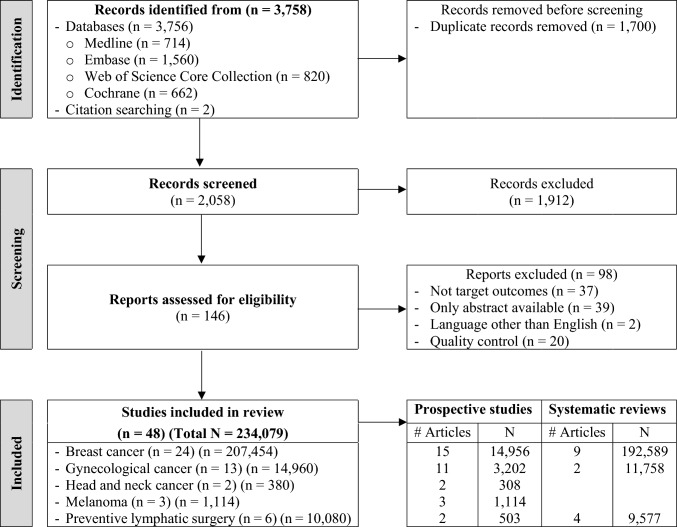
Flow diagram of search results

A literature review was performed in two phases. The first phase was conducted by a research librarian (DPF) who searched in the following four databases: MEDLINE, Embase, Web of Science Core Collection, and Cochrane Library. Publications dated between 2015 and 2023 were included, to gather entries released since previously published reviews on the topic [9, 18]. Using controlled vocabularies adapted to each respective database (Mesh; Emtree), search strategies were further refined, using the main keywords “lymphedema,” “cancer,” and “prospective studies.” We excluded publications on non-cancer-related lymphedema, animal studies, letters, small case series, case reports (*n* < 50), and those written in a language other than English. A search hedge was adapted for the prospective studies entry material [19]. The initial search yielded 3756 entries (Fig. 1).

In the second phase of the review, initial results were uploaded to Covidence, a web-based software platform that streamlines the production of reviews [20]. After we excluded duplications (*n* = 1700), titles and abstracts of 2058 entries were screened by two reviewers (MEL and SS), yielding a total of 146 studies. Two independent reviewers (MEL and DT) then performed a detailed review of these studies in accordance with predefined inclusion criteria. We required prospective assessment of CRL as a primary or secondary outcome and a minimum sample size of 50 patients. In keeping with the main goal of estimating CRL incidence, we also included retrospective reviews presenting prospective collection of lymphedema-related data. We therefore retained 68 studies.

### Quality control

Of the 13 JBI critical tools available, checklists for case controls [21], prevalence studies [22], randomized controlled trials [23], and systemic reviews [24] were utilized to assess these 68 studies. Each checklist concludes with a decision as to whether to include, exclude it, or seek additional information. Two of four reviewers (MEL, MI, AT, DT) independently evaluated each study. In case of disagreement, a third reviewer (MEL or MI) acted as an arbitrator. This process yielded a final total of 48 studies.

### Statistical analysis

The choice of methods for summary and subgroup analysis was limited by the heterogeneity of the data due to the absence of a standardized clinical lymphedema definition, variations in measurement techniques and length of follow-up. When feasible, we conducted subgroup analyses based on cancer type. Predefined review characteristics for analysis included sample size, type of lymphedema assessment (objective and subjective), measurement methods, and length of follow-up. Radiation therapy and lymph node dissection variables were also considered, when data were available.

## Results

The search of the four medical indices yielded a total of 48 articles (33 prospective studies and 15 systematic reviews), providing a total sample size of 234,079 cancer survivors (Fig. 1). Our search encompassed papers reporting on CRL according to any cancer type. However, only four categories of cancer were included breast, gynecological and urological, head and neck (H&N) and melanoma. A fifth category represented preventive lymphatic surgery aiming to reduced CRL incidence including lymphovenous anastomosis (LVA) and axillary reverse mapping (ARM).

Breast cancer studies were the most numerous (50%, *n* = 24) and provided 88.6% of the total study population. Gynecological and urological cancers represented 27.1% (*n* = 13) of the included articles, and 6.1% of the population. This was followed by preventive lymphatic surgery (12.5%, *n* = 6), mainly focussing on breast cancer patients, representing 4.3% of the total population. Melanoma and H&N cancers represented the lowest number of included articles with 6.3 and 4.2%, respectively, corresponding to 0.5 and 0.2% of the total population.

A total of nine clinical definitions were identified, whereby 43.8% of the studies used a difference of greater than 5–10% between the limbs to define lymphedema (Table 1). Additionally, some studies also used more than one definition when various methods of diagnosis were used. About a quarter of the studies (13 studies, 27%) did not specify their clinical definition of lymphedema.

**Table 1:** Lymphedema clinical definitions

Cancer site	# Studies	Volume			Circumference		BIS change	Clinical judgement	Self-reported	Other	Not specified
		> 5–10%	> 15–20%	> 200 ml	> 2 cm	> 3–4 cm					
Breast	24	13	1	2	7		3	2	5	2	7
Uro-gynecologic	13	4				1	2		6	3	3
H&N	2							2		1	
Melanoma	3	2	1						1	1	
PLS	6	2	1	1	3	1	1		1		3
Total	48	21	3	3	10	2	6	4	13	7	13

*BIS* Bio-impedance spectroscopy; *PLS* preventive lymphatic surgery

Our review yielded a plethora of diagnostic methods, grouped into seven different methods, whereby most studies (30 studies, 62.5%) used circumferences and self-reported (16 studies, 33.3%) methods (Table 2). Several studies used more than one method of diagnosis, while two studies did not report diagnostic methods.

**Table 2:** Diagnostic methods for lymphedema

Cancer site	# Studies	Circumference	Perometry	Water displacement	BIS	Self-Reported	Clinical examination	Other	Not reported
Breast	24	16	8	3	8	8	2	1	1
Uro-gynecologic	13	7			2	6	2	3	1
H&N	2							4	
Melanoma	3	1	2	1		1			
PLS	6	6	1	4	2	1	1	1	
Total	48	30	11	8	12	16	5	9	2

*BIS* Bio-impedance spectroscopy; *PLS* preventive lymphatic surgery

Subsequently, while extracting data, we were able to identify several trends regarding risk factors that might influence CRL incidence. Our findings suggest that the following could increase the risk of developing CRL: extensive surgery (e.g., tumor and/or lymph node dissection), increasing age, having received adjuvant therapy (both chemotherapy and radiation therapy), body mass index (BMI) greater than 25 or 30 at surgery and increasing post-treatment, insufficient physical activity levels, and post-surgical complications (e.g., lymphocele, wound infection, seroma). Conversely, education (preoperative and general about lymphedema), prospective surveillance with early identification of signs and symptoms, lymphedema risk reduction practices, weight management or weight reduction, scar tissue release and omission of axillary dissection, show a trend toward decreasing the risk of CRL.

### Breast cancer

A total of 15 prospective studies reporting on the incidence of breast cancer-related lymphedema (BCRL), representing nearly 15,000 patients, were identified (Table 3) [25–39]. Incidence findings were reported using either objective measures (limb circumference, Bio-Impedance Spectroscopy (BIS), perometry, Indocyanine Green (ICG)), or by self-report. Length of follow-up varied between 6 and 120 months.

**Table 3:** Studies reporting on BCRL incidence

Reference	*N*	Lymphedema incidence (95% CI) *n* at risk (LFU %)						
		6 months	12 months	18 months	24 months	30–36 months	44–60 months	120 months
Armer 2019 [25]	488	4.3% (3.0–7.3) 370 (LFU 24%)	30.7% (26.4–35.8) 241 (LFU 64%)	45.0% (40.1–50.5) 175 (LFU 64%)	53.9% (48.8–59.5) 126 (LFU 74%)	60.3% (55.0–66.2) 63 (LFU 87%)		
	Self-R	2.1%	13.6%	23.2%	27.8%	30.9%		
Bundred 2020 [26]	1100	10.5% 928 (LFU 16%)	15.6% 899 (LFU 24%)	19.3% 777 (LFU 50%)	22.4% 545 (LFU 74%)	26.3% 314 (LFU 71%)	31.9% 156 (LFU 86%)	
	Self-R	42.9%	56.9%	62.1%	65.8%	69.3%	74.3%	
Isik 2022 [27]	2940				2.0%			
Kilbreath 2016 [28]	450			10.2%				
Kim 2015 [29]	313						42.2%	
Koelmeyer 2022 [30]	918					7.5%		
McDuff 2018 [31]	2266				7.1% 1436 (LFU 37%)		13.7% 398 (LFU 82%)	
Miller 2016 [32]	616				10.6% (8.4–13.7)			
Naoum 2020 [33]	1815						9.5%	
Pereira 2017 [34]	964				13.5% 890 (LFU 8%)		30.2% 525 (LFU 46%)	41.1% 216 (LFU 78%)
Salinas-Huertas 2022 [35]	201					13.9%		
Swaroop 2015 [36]	1121				5.3% (4.1–6.8)			
Terada 2020 [37]	631						9.2%	
	Self-R						20.4%	
Wetzig 2017 [38]	813						3.3%	
Zhu 2017 [39]	320					27.5%		
Total: 15	14,956	4.6–10.5%	15.6–30.7%	10.2–45.0%	2.0–53.9%	7.5–60.3%	3.3–31.9%	41.1%
	Self-R	2.1–42.9%	13.6–56.9%	23.2–62.1%	27.8–65.8%	30.9–69.3%	20.4–74.3%	

Reference	Measurement technique (Lymphedema definition)				Intervention			
	Circumference	BIS	Perometry	Other	SLNB	ALND	Chemotherapy	Various
Armer 2019 [25]	Volume ↑ > 10% or circumference ↑ > 2 cm			LBCQ			NAC	
Bundred 2020 [26]		↑ 2–3 standard deviation	RAVI > 10%	LBCQ				
Isik 2022 [27]			Not specified	ICG	1.4%/R: 1.0%	3.7%/R: 2.9%		
Kilbreath 2016 [28]	Not specified	Ratio exceed normative-based or ↑ > 0.1 from baseline			3.3%	18.2%		
Kim 2015 [29]	Volume change > 5%	Volume change > 5%					NAC	
Koelmeyer 2022 [30]	Not defined	Not defined						
McDuff 2018 [31]			RAVI ↑ > 10%		24 months: 3.7%/R: 4.3% 60 months: 8.3%/R 12.2%	24 months: 12.7%/R: 19.0% 60 months: 24.6%/R: 31.2%		
Miller 2016 [32]			RAVI ↑ > 10%					IR: 5.1% I: 4.1% A: 9.9% NoR: 26.7%
Naoum 2020 [33]			RVC ≥ 10%		8.0%/R: 10.7%	24.9%/R: 30.1%		
Pereira 2017 [34]	Difference 200 ml							
Salinas-Huertas 2022 [35]	Excess > 10%				4.6%	31.0%		
Swaroop 2015 [36]			RVC ≥ 10%				T: 10.3% NT: 4.9% NoC: 3.1%	
Terada 2020 [37]	Difference ≥ 2 cm 1 or more points			PRO-CTCAE	1.4%	24.1%		
					4.1%	51.8%		
Wetzig 2017 [38]	% change from baseline				1.7%	5.0%		
Zhu 2017 [39]	↑ > 5%						T: 32.1% NT: 19.9%	
Total	9	5	6	4	7 (1.0–12.2%)	10 (2.9–60.3%)	4 (4.6–60.3%)	4
					1 (4.1%)	3 (2.1–74.3%)	1 (2.1–30.9%)	

Note: Variation and 95% CI are provided when reported by the study

↑: Increase; *A* Autologous, *ALND* axillary lymph node dissection, *BIS* bio-impedance spectroscopy, *CI* confidence interval, *I* Implant, *ICG* indocyanine green, *IR* immediate reconstruction; *LBCQ* lymphedema breast cancer questionnaire, *LFU* lost to follow-up; *NAC* neo-adjuvant chemotherapy; *NoC* no chemotherapy; *NoR* no reconstruction, *NT* non-taxane, *PRO-CTCAE* patient-reported outcomes version of the common terminology criteria for adverse events, *R* regional lymph node radiation, *RAVI* relative arm volume increase, *RVC* relative volume change, *Self-R* Self-Report, *SLNB* sentinel lymph node biopsy, *T* taxane

For objective measures, BCRL incidence ranged from 2 to 60.3%. While the majority of studies (9 studies) utilized circumferential measurements to assess for lymphedema, variations in how lymphedema was defined were noted. Lowest incidence was reported with perometry and ICG at 24 months. For self-report, BCRL incidence ranged from 2.1 to 74.3%. Survivors treated with axillary lymph node dissection (ALND), chemotherapy and/or regional lymph node radiation reported a higher incidence of BCRL, varying between 2.9% (perometry and ICG) and 60.3% (circumference). Table 3 further highlights the heterogeneity of the findings reported for the breast cancer population.

Additionally, nine systematic reviews reporting on BCRL incidence met our inclusion criteria, encompassing a total of 283 articles and representing over 192,000 BCRL patients (Table 4) [40–48]. Some systematic reviews provided pooled lymphedema estimates, varying between 4 and 23.6%, and/or a variation in lymphedema incidence ranging from 0% to 63.4%.

**Table 4:** Systematic reviews reporting on BCRL incidence

Reference	*N* (# studies)	Sub-group	Lymphedema definition	Lymphedema measurement	Length of follow-up/Intervention	Lymphedema Incidence	
Reference included^a^						Pooled (95% CI)	Variation
Bakri 2022 [40] Wetzig 2017 [38]	(38 studies)	3515 1971 5288 3866 491 3136 657 762	Not specified	Not specified (objective measures)	ALND < 12 months ALND 12–24 months ALND > 24 months SLNB < 12 months SLNB 12–24 months SLNB > 24 months ARM + ALND < 12 months ARM + ALND > 12 months	16.5% (11–22) 24.6% (11–38) 23.6% (16.4–30.9) 7.5% (4.9–10.1) 3.7% (1.8–5.6) 5.9% (3.6–8.1) 3.3% (1.9–4.7) 6.4% (1.9–10.9)	
Gebruers 2015 [41]	9588 (28 studies)		Not specified	Circumference, water displacement, subjective	Circumference Water displacement PRO 6 months 12 months 18 months > 18 months		1–63.4% 0–15.8% 0–11% 2.0–10% 3.0–63.4% 6.6–7% 6.9–8.2%
Lin 2021 [42] Kilbreath 2016 [28] McDuff 2018 [31]	20,312 (19 RCTs)		↑ > 2 cm, ↑ ≥ 10%	Circumference, self-reported, arm volume	60 months (10.5–160 months)	14.3% (13.8–14.8)	3.0–36.7%
Pilger 2021 [43]	4110 (9 RCTs)		↑ circumference, ↑ > 2 cm, ↑ > 10% or 15%,	Circumference (volume), self-reported	6 months 12 months 24 months		0–11% 4–15% 1–14%
Rafn 2022 [44] Bundred 2022 [26]	3545 (22 studies)	1527	Not specified	BIS, physician diagnosis	Restricted to ALND	4% 6%	
Shah 2021 [45] Armer 2019 [25] Swaroop 2015 [36] Wetzig 2017 [38]	67,712 (50 studies)		↑ > 10%, L-DEX ratio > 7, ↑ > 2 cm, subjective symptoms	BIS, circumference	Background Circumference BIS	12.9% (5.1–10.0) 17% (10.3–23.7) 3.1% (1.3–4.9)	
Shaitelman 2017 [46]	14,373 (21 studies)	4379 1882 3115 2895 2102	Not specified	Perometry, circumference, water displacement, self-report	All Breast/CW Breast/CW + SCV Breast/CW + SCV + PAB Breast/CW + SCV + IMN Breast/CW + SCV + PAB + IMN	11% 7.4% (5.1–10.0) 15.5% (8.0–23.0) 12.2% (6.8–17.6) 10.8% (9.7–12.0) 13.5% (5.4–24.4)	
Shen 2023 [47] Bundred 2022 [26] Kilbreath 2016 [28] Kim 2015 [29] Naoum 2020 [33] Pereira 2017 [34] Swaroop 2015 [36] Zhu 2017 [39]	58,358 (84 studies)		Interlimb difference > 2 cm or 10%, RVC ≥ 200 ml or 10%, clinical diagnosis, self-report	Circumference, water displacement, BIS, perometry, self-reported, clinical observation	All (3 to 290 months) Circumference Water displacement BIS Perometry Self-report Both objective and subjective	21.9% (19.8–24.0)	4.5–58.8% 4.5–42.2% 11.7–22.2% 5.0–31.9% 7.2–54.0% 11.6–54.0% 5.1–58.8%
Wu 2019 [48]	8039 (12 studies)		Interlimb difference ≥ 2 cm	Circumference, self-reported	All (14.9 months–20 years)	26.2%	
Total: 9	192,589^a^					3.1–26.2%	0–63.4%

Note: Variation and 95% CI are provided when reported by the study

↑: Increase, *ALND* axillary lymph node dissection, *ARM* axillary reverse mapping, *BIS* bio-impedance spectroscopy, *CI* confidence interval, *CW* chest wall, *IMN* internal mammary nodes, *PAB* posterior axillary boost; *PRO* patient-reported outcomes, *RCT* randomized controlled trial, *RVC* relative volume change, *SCV* supraclavicular fossa, *SLNB* sentinel lymph node biopsy

^a^*n* from included references were excluded from total N

Some systematic reviews presented lymphedema incidence based on the measurement technique used. Water displacement tends to report the lowest incidence (0–22.2%), followed by BIS (3.1–31.9%), self-report (0–54.0%), perometry (7.2–54.0%), and circumferences measurements (1–63.4%). As observed in prospective studies, ALND showed a higher incidence in the systematic reviews varying from 6 to 24.6%, compared to 3.7% to 7.5% for SLNB.

### Gynecological and urological cancers

Eleven studies, with over 3200 patients, representing cervical, endometrial, ovarian, and vulvar malignancies were included (Table 5) [49–59]. The length of follow-up varied between 6 and 120 months, with the highest incidence of 45.1% self-reported by patients at 24 months. Incidence varied among the different types of gynecological cancers, with all of them reporting a lower incidence when neither lymph node dissection nor sentinel lymph node biopsy (SLNB) were performed. The highest incidence was reported by women treated for vulvar (66.7%), followed by cervical (51.7%), endometrial (44.4%), and ovarian (40.4%) cancer. Most studies assessing for lower limb lymphedema (LLL) post-gynecological cancers used circumferential measurements as an objective measure (Table 5).

**Table 5:** Studies reporting on gynecological CRL incidence

Reference	*N*	Lymphedema incidence (95% CI) *n* at risk (LFU %)							
		6 months	6–12 months	12–18 months	15–24 months	24 months	> 37 months	60 months	120 months
Carlson 2020 [49]	914					34.2%			
Cibula 2021 [50]	150	13.6%	17.8%		25.1%	27.2%			
	Self-R					10.7%			
Geppert 2018 [51]	188		12.7% 181 (LFU 3.7%)						
Hareyama 2015 [52]	358		12.9%					20.3%	25.4%
Hayes 2017 [53]	217		30.4% 194 (LFU 11%)		37.3% 217				
	Self-R 339		35.6% 331 (LFU 2%)		45.1% 339				
Ki 2016 [54]	413						11.1%		
Mathevet 2021 [55]	206	41.3%							
Pigott 2020 [56]	171		28.8% 39 (LFU 77%)		33.3% 45 (LFU 74%)				
	Self-R 227		34.9% 166 (27%)		45.0% 171 LFU (25%)				
Ritchie 2022 [57]	Self-R 75							12%	
Watson 2019 [58]	97		19% 63 (LFU 35%)	27% 55 (LFU 43%)					
Wedin 2020 [59]	235		9.4%						
	Self-R		7.7%						
Total: 11	3202	13.6–41.3%	12.7–30.4%	27%	25.1–37.3%	27.2–34.2%	11.1%	20.3%	25.4%
	Self-R		7.7–35.6%		45.0–45.1%	10.7%		12.0%	

Reference	Measurement technique (LE definition)			Intervention			
	Circumference	BIS	Other	Cervical	Endometrial	Ovarian	Vulvar
Carlson 2020 [49]	≥ 10%			34.8% (*n* = 138)	33.7% (*n* = 734)		42.9% (*n* = 42)
Cibula 2021 [50]	Volume change		Patient-perceived swelling	SLNB			
				10.7%			
Geppert 2018 [51]			CTCG (> 5%)		SLNB: 1.3% TLND: 21.0%		
Hareyama 2015 [52]			Gynecologist (ISL grading)	*n* = 100	*n* = 121	*n* = 137	
Hayes 2017 [53]		Ratio arm/leg ≥ 1 SD	Patient self-report of swelling	6–12 mo: 25.0% 15–24 mo: 33.3% (*n* = 24)	6–12 mo: 34.8% 15–24 mo: 42.4% (*n* = 125)	6–12 mo: 27.5% 15–24 mo: 33.9% (*n* = 56)	6–12 mo: 9.1% 15–24 mo: 8.3% (*n* = 12)
				6–12 mo: 46.2% 15–24 mo: 51.7% (*n* = 29)	6–12 mo: 34.7% 15–24 mo: 44.4% (*n* = 198)	6–12 mo: 29.8% 15–24 mo: 40.4% (*n* = 94)	6–12 mo: 61.1% 15–24 mo: 66.7% (*n* = 18)
Ki 2016 [54]	Not specified		US/MRI			11.1%	
Mathevet 2021 [55]	NCI-CTCAE			SLNB: 31.4% TLND: 51.5%			
Pigott 2020 [56]		Predictive equation	SRLS				
Ritchie 2022 [57]			GCLQ (GCLQ criteria)				
Watson 2019 [58]	↑ volume > 10%				6–9 months SLNB: 17% (*n* = 29) TLND: 19% (*n* = 26) 12–18 months SLNB: 25% (*n* = 28) TLND: 24% (*n* = 21)		
Wedin 2020 [59]	↑ relative volume > 10%		LYMQOL		NoLND: 3.4% TLND: 15.8%		
					NoLND: 5.1% TLND: 10.7%		
Total	6	2	7	5 (13.6–51.5%)	8 (1.3–42.4%)	3 (11.1–33.9%)	2 (8.3–42.9%)
				2 (10.7–51.7%	2 (5.1–44.4%)	1 (29.8–40.4%)	1 (61.1–66.7%)

Note: Variation and 95% CI are provided when reported by the study

↑: Increase, *BIS* bio-impedance spectroscopy, *CI* confidence interval, *CTCG* common toxicity criteria grading, *GCLQ* gynecological cancer lymphedema questionnaire, *ISL* International Society of Lymphology, *LE* lymphedema;LFU: Lost to Follow-up, *LYMQOL* lymphedema specific QOL questionnaire; *mo* months, *MRI* magnetic resonance imaging, *NCI-CTCAE* National Cancer Institute Common Toxicity Criteria; *NoLND* no lymph node dissection, *SD* standard deviation; *Self-R* self-report; *SLNB* sentinel lymph node biopsy, *SRLS* self-report leg swelling, *TLND* total lymph node dissection, *US* ultrasound

One systematic review on prostate cancer [60] and one on vulvar cancer [61] were included with a total sample size of 11,758 patients (Table 6). For prostate cancer, Clinckaert et al. (2022) [60] reported LLL varying from 0 to 29%, and genital lymphedema varying from 0 to 22%, respectively. A higher incidence was found in those who underwent pelvic lymph node dissection (PLND) and radiotherapy. Huang et al. (2017) [61] provided a pooled LLL estimate of 28.8% in vulvar malignancies, with the highest incidence in cross-sectional studies (49.2%) or randomized controlled trials (45.1%).

**Table 6:** Systematic reviews reporting on gynecological and urological CRL incidence

Reference (Cancer type)	*N* (# studies)	Sub-group	Lymphedema definition	Lymphedema measurement	Length of follow-up/Intervention	Lymphedema incidence	
						Pooled (95% CI)	Variation
Clinckaert 2022 [60] (Prostate)	9223 (18 studies)		Not specified	Not specified	3–180 months Lower Limb Lymphedema - Prostatectomy + PLND - Radiation - PLND + Radiation Genital Lymphedema - Prostatectomy + PLND - Radiation - PLND + Radiation		0–29% 0–29% 0–14% 0–9% 18–29% 0–22% 0–1% 0–8% 2–22%
Huang 2017 [61] (Vulvar)	2535 (27 studies)	565 198 1606 166	22 studies not reported 2: > 3 cm 2: ↑ > 10% 1: self-report	Clinical diagnosis, circumference, self-report, lymphoscintigraphy	*Overall* Prospective RCT Retrospective Cross-sectional	28.8% 16.7% 45.1% 26.0% 49.2%	16.7–49.2%
Total: 2	11,758						0–49.2%

Variation and 95% CI are provided when reported by the study

↑: Increase; *CI* confidence interval, *PLND* pelvic lymph node dissection, *RCT* randomized controlled trial

### Head and neck cancers

A total of 380 participants, distributed in two studies were included (Table 7) [62, 63]. Participants of both studies received similar interventions. The incidence of H&N CRL tends to be higher in the early phase post-treatment, varying from 80 to 90.1%, while decreasing over time to 70.6–82.3%. Ridner et al. (2016) [62] reported that external lymphedema tends to vary between 81.9 and 90.1%, internal lymphedema between 80.4 and 89.4%, and a combination of both between 70.6 and 80.9%. Tribius et al. (2020) [63] reported an incidence of 80% between 3 and 6 months in advanced stage H&N cancer.

**Table 7:** Studies reporting on Head and Neck CRL incidence

Reference	*N*	Lymphedema incidence (95% CI) (*n* and LFU)			
		3–6 months	9 months	12 months	> 12 months
Ridner 2016 [62]	100				
- External lymphedema		90.1% (81.7–94.9) 81 (LFU 19%)	81.9% (71.5–89.1) 72 (LFU 28%)	85.5% (74.6–92.2) 62 (LFU 38%)	82.3% (70.9–89.8) 62 (LFU 38%)
- Internal lymphedema		85.7% (75.6–92.1) 70 (LFU 30%)	84.3% (71.9–91.8) 51 (LFU 49%)	89.4% (77.4–95.4) 47 (LFU 53%)	80.4% (67.5–89.0) 51 (LFU 49%)
- Both		80.9% (69.9–88.5) 68 (LFU 32%)	70.6% (57.0–81.3) 51 (LFU 49%)	76.1% (62.0–86.1) 46 (LFU 54%)	70.6% (57.0–81.3) 51 (LFU 49%)
Tribius 2020 [63]	280	80%			
Total: 2	380	80–90.1%	70.6–84.3%	76.1–89.4%	70.6–82.3%

Reference	Measurement technique (Lymphedema definition)				Intervention		
	ACSLHN	Endoscopy	Neck US	Patterson	Surgery	Radiation	Chemotherapy
Ridner 2016 [62]	ACSLHN staging criteria			Clinical judgement			
Tribius 2020 [63]		Clinical judgement					
Total	1	2	1	1	2	2	2

Note: Variation and 95% CI are provided when reported by the study

*ACSLHN* American Cancer Society Lymphedema Head and Neck, *CI* confidence interval; *LFU* lost to follow-up

### Melanoma cancers

The three melanoma studies all distinguished between upper and lower limb lymphedema (Table 8) [64–66]. The overall CRL incidence varied between 2 and 28.6%, with the lowest incidence attributed to upper limb or trunk melanoma treated with SLNB (1.0–18.4%), and the highest to lower limb or trunk melanoma treated with “total” lymph node dissection (TLND) (7.7–47.4%). Only one study [65] had participants self-reporting an incidence of 23.1% at a median time of 37 months after SLNB in both upper and lower limb CRL.

**Table 8:** Studies reporting on Melanoma CRL incidence

Reference	*N*	Lymphedema incidence (95% CI) (*n* and LFU)			
		3–6 months	9–12 months	15–18 months	> 36 months
Cromwell 2015 [64]	277	21.7% 244 (LFU 12%)	25.9% 197 (LFU 29%)	28.6% 126 (LFU 55%)	
Morton 2017 [65]	694				2.0%
	Self-R				23.1%
Nacchiero 2019 [66]	143				21.0%
Total: 3	1114	21.7%	25.9%	28.6	2–21.0%
	Self-R				23.1%

Reference	Measurement technique (Lymphedema definition)				Intervention			
	Circumference	Perometry	Water	Other	UL SLNB	UL ALND	LL SLNB	LL TLND
Cromwell 2015 [64]		Volume change > 10%			3–6 mo: 10.9% 9–12 mo: 18.4% 15–18 mo: 12.1% (*n* = 73)	3–6 mo: 19.1% 9–12 mo: 35.1% 15–18 mo: 36.8% (*n* = 76)	3–6 mo: 7.3% 9–12 mo: 10.8% 15–18 mo: 25.0% (*n* = 52)	3–6 mo: 42.3% 9–12 mo: 47.4% 15–18 mo: 38.7% (*n* = 76)
Morton 2017 [65]		Volume difference ≥ 10%		Self-R	1.0% (*n* = 411)		2.0% (*n*= 238)	
Nacchiero 2019 [66]	Sum circumference point ≥ 7% or % change ≥ 15%				13.7% (*n* = 51)	0.0%^a^ (*n* = 8)	31.9% (*n* = 69)	7.7%^a^ (*n* = 15)
Total	1	2	1	1	3 (1.0–18.4%)	2 (0.0–36.8%)	3 (2.0–31.9%)	2 (7.7–47.4%)

Note: Variation and 95% CI are provided when reported by the study

*ALND* Axillary lymph node dissection, *CI* confidence interval, *LFU* lost to follow-up, *LL* lower limb, *Self-R* self-report, *SLNB* sentinel lymph node biopsy, *TLND* total lymph node dissection, *UL* upper limb

^a^ALND was performed in combination with multiple LVA

### Preventive surgery

Two prospective studies [67, 68] and four systematic reviews [69–72] on surgery aiming to prevent lymphedema were included, representing a total of 10,080 patients (Table 9). Preventive surgery included LVA or ARM procedures, in cases where full lymph node dissection was judged to be required. The reported CRL incidence was 16–28.5% and 5.2–23.4% for prospective studies and systematic reviews, respectively. Patients receiving the preventive procedure experienced a lower incidence of CRL compared to controls in both prospective studies and systematic reviews: 3–21% versus 19–42%, 2–18% versus 14.1–48.5%, respectively.

**Table 9:** Studies and systematic reviews reporting on preventive surgery CRL incidence

Reference	N	Population	LE incidence (95% CI)	Measurement technique (Lymphedema definition)	Intervention	
			12–15 months/Pooled		Preventive	Control
*Prospective studies*						
Gennaro 2022 [67]	123	Breast	28.5%	Circumference (↑ ≥ 2 cm in one or more places)	21.0% (*n* = 62)	42.0% (*n* = 61)
Ozmen 2019 [68]	380	Breast	16.0%	Circumference (Difference ≥ 2 cm)	3.0% (*n* = 74)	19.0% (*n* = 306)
Total: 2	503		16.0–28.5%		2 (3.0–21.0%)	2 (19.0–42.0%)
*Systematic reviews (# studies)*						
Ciudad 2022 [69] (24 studies)	1547	Breast (*n* = 1247)	5.2% (2.9–7.5)	Circumference, volumetry, BIS, clinical, lymphoscintigraphy (Not specified)	7.6%° (*n* = 288)	22.6%° (*n* = 549)
		Gynecological (*n* = 300)	6.7% (< 1–13.4)		18.0%° (*n* = 50)	48.5%° (*n* = 132)
Co 2022 [70] (5 studies)	1639	Breast		Circumference, volumetry (Not specified)	4.8%° (*n* = 766)	18.8%° (*n* = 873)
Johnson 2019 [71] (19 studies)	3035	Breast	23.4%	Circumference, L-Dex, volumetry, perometry (Excess volume > 200 ml, ↑ > 5%-20, > 1–4 cm, abnormal L-DEX score, self-report)	2.1% (*n* = 48)	14.1% (*n* = 1419)
					RLNR: 10.3% (*n* = 58)	RLNR: 33.4% (*n* = 1510)
Wijaya 2020 [72] (29 studies) ^a^4/5 studies included in Co 2022, removed from total N	4954	Breast	7% (4–11)	Circumference, water displacement, self-report (Not specified)	SLNB: 2% (1–3) ALND: 14% (5–26) SLNB + ALND: 11% (1–30)	
Total: 4	9577^a^		5.2–23.4%		2–18.0%	14.1–48.5%

Note: Variation and 95% CI are provided when reported by the study

↑: Increase; *ALND* axillary lymph node dissection, *CI* confidence interval; *LE* lymphedema; *RLNR* regional lymph node radiation, *SLNB* sentinel lymph node biopsy

^a^Data extracted from randomized controlled trials

## Discussion

In terms of trends in the incidence of CRL, previous work from Cormier et al. (2010) [9] published an overall CRL incidence of 15% for all cancer site (melanoma, genitourinary, gynecological cancers, excluding BCRL). Shaitelman et al. (2015) [18] extended the work from Cormier et al. (2010) [9] and published in addition a pooled incidence of 6.3% for BCRL in patients who underwent SLNB, and 22.3% for BCRL in patients who underwent ALND. For the purpose of this updated review on incidence of CRL, given the heterogeneity of data pertaining to lymphedema definitions, diagnostic methods, and variations in length of follow-up, meta-analysis, and pooled incidence could not be conducted. Findings were therefore reported by cancer site to provide tentative inferences.

For BCRL post-ALND, Shaitelman et al. (2015) [18] reported objective incidences varying from 11 to 57%, compared with our findings of 3–60%. For SLNB alone, Shaitelman et al. (2015) [18] reported a rate of 0–23%, whereas our review narrowed the range to 1–12%, which might be attributed to the fact that most studies (4 studies, 57.1%) used perometry to assess BCRL. Our review was able to provide additional information in terms of a variation of 3–31% of BCRL incidence with radiation treatment, and 5–60% when chemotherapy is a modality of treatment. Therefore, the incidence of BCRL remains important, especially when combined with ALND, regional lymph node radiation and chemotherapy.

For gynecological cancers, previous reviewers reported an incidence rate of 0–73%, with the highest rate observed in vulvar cancer (0–73%), followed by cervical cancer (2–49%), and lastly for endometrial cancer (1%). Our review diverges significantly from these findings, revealing a notably higher rate for endometrial cancer (1–42%), along with a narrowed range for vulvar cancer (8–43%) and for cervical cancer (14–52%). The cumulative incidence rate for gynecological cancers also shows a reduced range, from 0 to 73% in previous reviews to 11–41% in our findings. Moreover, SLNB alone decreases the rate, with our results aligning with previous reviews showing a rate of 0–25%, compared to our 1–31%.

In terms of genitourinary cancer incidence, the two preceding reviews indicated a variation of 1–18% for prostate cancer, while our findings show a broader range of 0–29%. Additionally, those reviews covered penile and bladder cancer, reporting rates of 20–21% and 15–23%, respectively. Unfortunately, we encountered a lack of available studies to enable an update on the incidence for those two cancers.

For H&N cancers, our results differ markedly from the previously published reviews. Cornier et al. (2010) [9] and Shaitelman et al. (2015) [18] reported a range of 0–8%, whereas more recent studies suggest a rate of 80–90%. This concurs with our clinical experience.

For melanoma, there has been a significant reduction and narrowing of cumulative ranges. Previous studies indicated an overall range of 1–61%, whereas we report a range of 2–29%. While the ranges for lower extremity lymphedema following total lymph node dissection for melanoma have narrowed, they still remain substantial: 6–61% in previous reviews, compared to 8–47% in our findings. An unexpected finding is the substantial lymphedema rate after SLNB alone: 1–15% in previous reviews and 1–32% in our study.

Preventive lymphatic surgeries, such as LVA or ARM, performed at the same time of lymph node dissection are relatively recent innovations. Consequently, there was no previous comparison data on the incidence rates following these preventive procedures.

The existing data on lymphedema incidence remains primarily focused on the breast cancer population. A paucity of evidence persists for non-breast CRL, including gynecological (uterus, ovaries, cervix, or vulva), urological (prostate, bladder, urethral, kidney, testicular, and penile cancers), gastrointestinal (colorectal, anal, bile duct, pancreatic, gastric, and liver cancer), melanoma, H&N cancers, lymphoma, and sarcoma. Given that clinically we observe CRL in many of these patients, a collaborative effort is needed to capture its incidence in these populations that are underrepresented in research studies.

As concluded by Cormier et al. (2010) [9] and Shaitelman et al. (2015) [18], a significant challenge in lymphedema research lies in establishing consensus among researchers regarding diagnostic standards and measurement techniques. Researchers and clinicians use various objective tools and methodologies to diagnose lymphedema, such as clinical evaluation, circumferential measurements, bioimpedance, water displacement, perometry, imagery, and self-report measures. This diversity in diagnostic approaches contributes to a wide range of reported incidence rates, complicating comparisons between studies. Research settings using technology, such as BIS or perometry, may tend to report a lower lymphedema incidence. On the other hand, research settings using circumferential techniques for its ease and low cost of use, and more likely representative of clinical practice, are potentially reporting a higher incidence of lymphedema. Studies relying solely on one diagnostic method may underestimate the true incidence of lymphedema.

While objective tools are crucial for diagnosing lymphedema, self-reported symptoms such as swelling, sensation of heaviness, perceived limb size difference and discomfort play an equally vital role in initial diagnostic screening [73]. Healthcare professionals must attentively consider these patient self-reported symptoms, as they may facilitate early detection of lymphedema and lead to earlier management, reduced complications and financial burden, while improving a patient’s QOL.

## Conclusion

Our findings revealed significant CRL incidence across several cancer types. Early identification of lymphedema signs and symptoms and prompt referral to a certified lymphedema therapist are crucial to prevent the myriad complications resulting from inadequate management of this chronic and progressive condition. As patients increasingly survive cancer treatment, clinicians bear responsibility to minimize the burden on patient’s QOL from the multitude of potential side effects that may arise. To facilitate future research, the international lymphedema community should make it a priority to converge on accurate, reproducible, accessible, cost-effective, reliable, and quantifiable diagnostic methods. Such standardization efforts would enhance research quality and mitigate discrepancies in reported incidence rates in the future.
